# Designing a strain-tunable hydrogenated GeCH/Janus HfSO van der Waals heterostructure for solar-energy conversion

**DOI:** 10.1039/d6ra08066a

**Published:** 2026-10-23

**Authors:** Tran T. Nhan, Ngoc-Trung Nguyen, Trang Minh Pham, Tam Duy Nguyen, Huynh V. Phuc, Nguyen N. Hieu, Chuong V. Nguyen, Yoshiyuki Kawazoe, Thi Viet Bac Phung

**Affiliations:** a Faculty of Fundamental Sciences, Hanoi University of Industry 298 Cau Dien Hanoi 100000 Vietnam tran.nhan@haui.edu.vn; b Center for Environmental Intelligence, VinUniversity Hanoi 100000 Vietnam; c School of Chemistry, Monash University Melbourne Victoria 3800 Australia; d Division of Physics, School of Education, Dong Thap University Cao Lanh 870000 Vietnam; e Institute of Research and Development, Duy Tan University Da Nang 550000 Vietnam hieunn@duytan.edu.vn; f Faculty of Natural Sciences, Duy Tan University Da Nang 550000 Vietnam; g Faculty of Mechanical Engineering, Le Quy Don Technical University Hanoi 100000 Vietnam; h New Industry Creation Hatchery Center (NICHe), Tohoku University Sendai 980-8579 Japan; i Department of Centre for Inter Disciplinary Research, SRM University-AP Amaravati 522240 Andhra Pradesh India; j College of Engineering and Computer Science, VinUniversity Hanoi 100000 Vietnam bac.ptv@vinuni.edu.vn

## Abstract

The rational integration of complementary two-dimensional (2D) semiconductors into van der Waals (vdW) heterostructures provides an effective route for engineering interfacial electronic and optoelectronic properties. Herein, we systematically investigate the GeCH/HfSO vdW heterostructure by first-principles calculations, with particular emphasis on the roles of interfacial stacking, Janus polarization, and biaxial strain. The optimized heterostructure exhibits weak vdW interlayer coupling, together with dynamical and thermal stability. The electronic structure reveals a type-II band alignment, with the valence-band maximum predominantly localized on GeCH and the conduction-band minimum on HfSO. The heterostructure also exhibits enhanced broadband optical absorption with coefficients on the order of 10^5^ cm^−1^. More importantly, biaxial strain enables effective control of the band alignment, inducing a transition from type-I to type-II, while tensile strain continuously enhances the theoretical estimated photovoltaic conversion efficiency from 2.23% at zero strain to 28.60% at *ε* = +10%. These results establish GeCH/HfSO as a mechanically tunable vdW heterostructure with strong interfacial charge-separation capability and promising potential for next-generation optoelectronic and solar-energy conversion applications.

## Abstract 2

The rational integration of complementary two-dimensional (2D) semiconductors into van der Waals (vdW) heterostructures provides an effective route for engineering interfacial electronic and optoelectronic properties.

## Graphical abstract

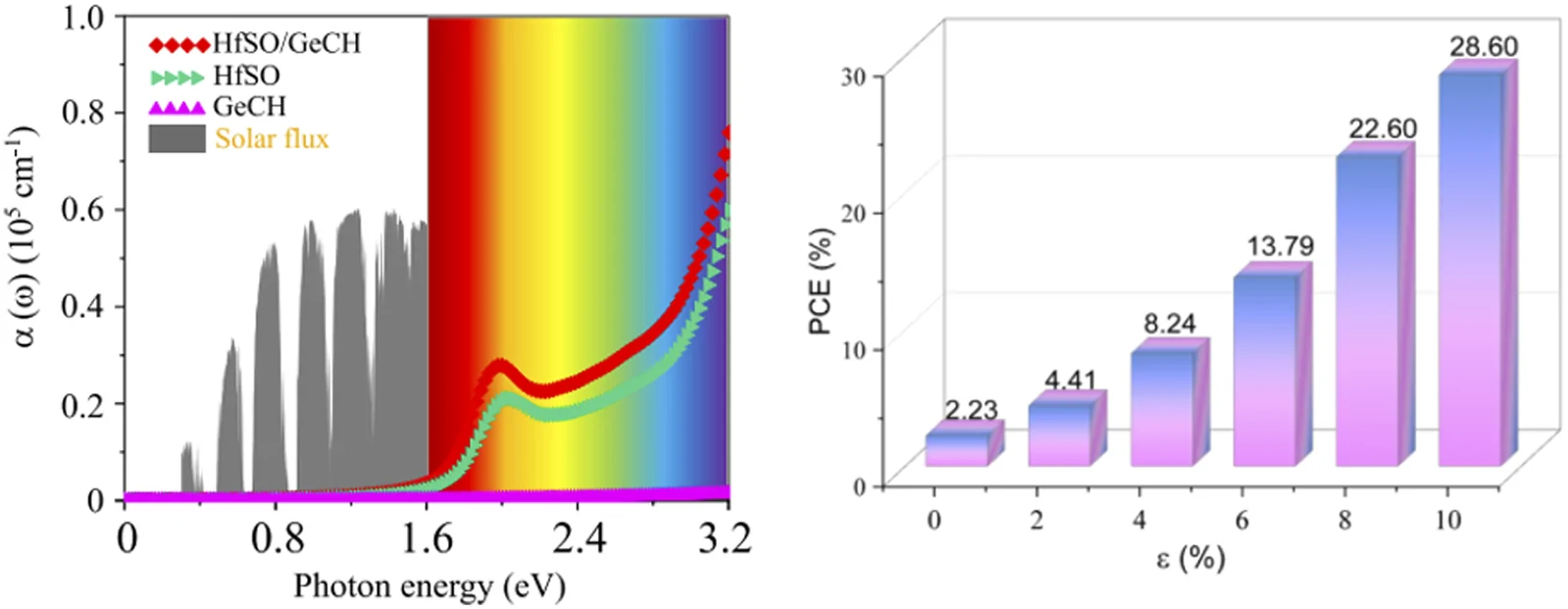

## 1 Introduction

The continuous expansion of the two-dimensional (2D) material family^1–5^ has shifted current research from the exploration of individual monolayers toward the rational design of multifunctional hybrid systems. Rather than relying on the intrinsic characteristics of a single atomic layer, constructing van der Waals (vdW) heterostructures^6–10^ offers an effective strategy for tailoring material behavior through interfacial interactions while largely preserving the structural integrity of each constituent. The weak interlayer coupling enables the integration of chemically and structurally distinct 2D crystals without the stringent lattice-matching requirements commonly encountered in conventional epitaxial growth.^11^ More importantly, the electronic states near the interface can be redistributed through charge transfer, electrostatic polarization, and orbital hybridization, leading to emergent properties that are unattainable in the isolated components.^12–14^ Such interface-driven phenomena have established vdW heterostructures as a versatile platform for engineering electronic structures and optimizing functional performance in multifunctional and energy-conversion technologies.

Particularly, vdW heterostructures composed of two semiconducting monolayers have emerged as an effective platform for tailoring interfacial electronic properties beyond those attainable in isolated materials.^15–17^ The electronic behavior of these heterostructures is primarily determined by the relative alignment of the conduction- and valence-band edges of the constituent layers, allowing the rational modulation of charge redistribution and carrier transport across the interface. Depending on the band-edge arrangement, semiconductor heterostructures can exhibit type-I, type-II, or type-III band alignment, each offering distinct functionalities for electronic and optoelectronic applications.^18–20^ Among these, type-II heterostructures are particularly attractive because the staggered band alignment promotes spontaneous spatial separation of photogenerated electrons and holes, thereby suppressing carrier recombination and prolonging carrier lifetime, which are highly desirable for photovoltaic and photocatalytic processes.^21,22^ Consequently, the exploration of semiconducting monolayers with compatible lattice structures and complementary electronic characteristics has become a key strategy for constructing high-performance vdW heterostructures.

To meet these requirements, functionalized group-IV monolayers^23^ have recently attracted increasing attention owing to their excellent structural stability and tunable electronic properties. Surface functionalization has proven to be an effective approach for stabilizing 2D materials while simultaneously tailoring their electronic structures for practical applications.^24–27^ Among them, the graphene-like GeCH monolayer has been identified as a promising semiconductor because of its thermodynamic stability, intrinsic semiconducting nature, and suitable band-edge positions.^28,29^ Complementary to the characteristics of GeCH, Janus 2D materials have recently emerged as an attractive class of semiconductors because their broken out-of-plane symmetry introduces physical properties that are absent in conventional centrosymmetric monolayers.^30,31^ Recently, the Janus HfSO monolayer has attracted increasing attention owing to its intrinsic vertical polarization, semiconducting nature, and favorable structural stability.^32,33^ The asymmetric distribution of sulfur and oxygen atoms across the Hf atomic layer generates a built-in electrostatic field, which can effectively regulate interfacial charge redistribution and facilitate the spatial separation of photogenerated carriers.^34^ Indeed, first-principles investigations have shown that HfSO-based vdW heterostructures exhibit favorable band alignment, efficient charge separation, and enhanced optical absorption, highlighting the potential of HfSO as a versatile building block for optoelectronic and photovoltaic applications.^35–37^

Despite the promising characteristics of GeCH and Janus HfSO as individual semiconducting monolayers, their interfacial compatibility and the physical properties arising from their integration into a vdW heterostructure remain largely unexplored. In particular, it is still unclear whether the GeCH/HfSO heterostructure can simultaneously achieve favorable structural stability, appropriate band alignment, efficient interlayer charge transfer, and enhanced optical response, all of which are essential for high-performance optoelectronic and photovoltaic applications. A comprehensive understanding of these interface-governed phenomena is therefore required to assess the feasibility of this previously unexplored heterostructure and to elucidate the relationship between atomic-scale interactions and its electronic and optical characteristics. Motivated by these considerations, we systematically investigate the structural, electronic, interfacial, optical and photovoltaic properties of the GeCH/HfSO vdW heterostructure using first-principles calculations.

## 2 Computational methods

All calculations were carried out using first-principles density functional theory (DFT) within the Vienna *Ab initio* Simulation Package (VASP).^38,39^ The electron-ion interaction was represented by the projector augmented-wave (PAW) formalism,^40^ whereas the exchange–correlation effects were described using the Perdew–Burke–Ernzerhof (PBE)^41^ functional under the generalized gradient approximation (GGA).^42^ The electronic wave functions were expanded in a plane-wave basis with a kinetic-energy cutoff of 500 eV. Geometry optimization was performed until the residual force acting on each atom was below 0.01 eV Å^−1^, and the total-energy variation between successive ionic steps was less than 10^−6^ eV. To eliminate artificial interactions arising from the periodic boundary condition, a vacuum region exceeding 30 Å was introduced perpendicular to the monolayer. Reciprocal-space integrations were carried out using a *Γ*-centered Monkhorst–Pack *k*-point grid with an 12 × 12 × 1 sampling density. Since the interaction between the GeCH and HfSO layers is dominated by weak dispersion forces, long-range vdW interactions were incorporated through the Grimme DFT-D3 (ref. 43) correction with Becke–Johnson (BJ) damping during structural relaxation. Considering the intrinsic out-of-plane asymmetry of the Janus layer and the GeCH/HfSO heterostructure, dipole correction was applied along the vacuum direction to eliminate artificial electrostatic interactions arising from the periodic boundary conditions. To improve the description of the electronic structure beyond the conventional GGA approximation, additional calculations were performed using the screened Heyd–Scuseria–Ernzerhof (HSE06) hybrid functional with Hartree–Fock exchange energy fraction of 0.25.^44^ Phonon dispersion relations were calculated using density functional perturbation theory (DFPT) implemented in the PHONOPY package^45^ to confirm the absence of imaginary vibrational modes. Thermal stability was assessed by *ab initio* molecular dynamics (AIMD) simulations^46^ within the canonical (NVT) ensemble regulated by a Nosé–Hoover thermostat. The simulations were conducted at 300 K for 8 ps with a time increment of 1 fs.

## 3 Results and discussion

Prior to constructing the GeCH/HfSO vdW heterostructure, the structural, electronic, and dynamical properties of the individual monolayers were systematically examined to verify their suitability as constituent layers. The optimized atomic structures of Janus HfSO and GeCH are presented in Fig. 1(a) and (b), respectively. The Janus HfSO monolayer adopts a typical S–Hf–O trilayer configuration, in which sulfur and oxygen atoms occupy opposite sides of the Hf atomic plane. Such an asymmetric atomic arrangement breaks the out-of-plane mirror symmetry, giving rise to an intrinsic vertical dipole moment and a built-in electrostatic field. In contrast, the optimized GeCH monolayer consists of a buckled Ge–C honeycomb framework with hydrogen atoms bonded to both Ge and C atoms on opposite surfaces, resulting in a fully hydrogenated configuration. The optimized lattice constants of both monolayers are *a*_HfSO_ = 3.30 Å and *a*_GeCH_ = 3.28 Å, which agree well with previously reported theoretical results.^29,32^ The electronic band structures obtained using the PBE and HSE06 functionals are displayed in Fig. 1(c) and (d). The results reveal that the two constituent monolayers exhibit different band-gap characteristics. Specifically, Janus HfSO is predicted to be a direct-gap semiconductor, with both the valence-band maximum (VBM) and conduction-band minimum (CBM) located at the *Γ* point. In contrast, the GeCH monolayer possesses an indirect band gap, where the VBM remains at the *Γ* point while the CBM is located at the *M* point. As expected, the HSE06 functional predicts larger band-gap values than the PBE approximation while preserving the overall band topology. The calculated band gaps of Janus HfSO monolayer are 1.23 eV (PBE) and 2.03 eV (HSE06), whereas those of GeCH monolayer are 3.72 eV and 4.67 eV, respectively. As shown in Fig. 1(c) and (d), the projected band structures obtained using the PBE and HSE06 functionals exhibit nearly identical band dispersions. The primary difference lies in the upward shift of the conduction-band states, leading to a significant increase in the band-gap values while leaving the valence-band features almost unchanged. Furthermore, the dynamical stability of these isolated monolayers was further evaluated through phonon dispersion calculations, as shown in Fig. 1(e) and (f). No imaginary phonon modes are observed throughout the entire Brillouin zone for either Janus HfSO or GeCH monolayers, confirming that both monolayers are dynamically stable.

**Fig. 1:**
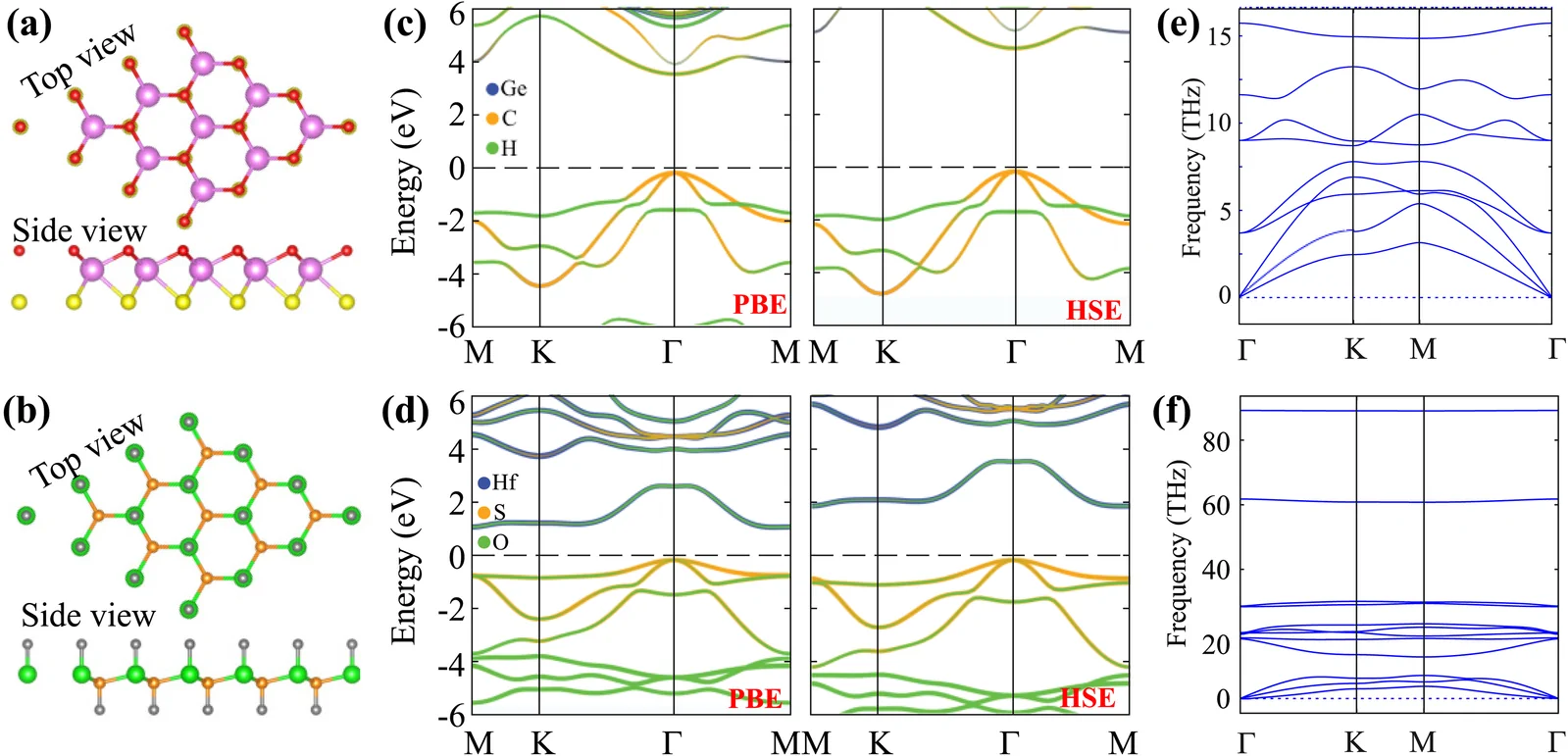
Optimized atomic structures of (a) Janus HfSO and (b) GeCH monolayers. Electronic band structures calculated using the PBE and HSE06 functionals for (c) Janus HfSO and (d) GeCH. Phonon dispersion spectra of (e) Janus HfSO and (f) GeCH monolayers.

Furthermore, to explore the interfacial characteristics of the proposed GeCH/HfSO system, different vdW heterostructures were designed by combining Janus HfSO and GeCH monolayers through vertical stacking. Considering the hexagonal symmetry of both constituent layers and the broken out-of-plane symmetry of the Janus HfSO layer, three representative stacking families, denoted as AA, AB, and AC, were examined, as illustrated in Fig. 2. Within each stacking family, four different stacking patterns were further considered according to the relative position of the sulfur or oxygen atoms in the Janus layer with respect to the GeCH monolayer. Consequently, the AA and AC configurations each contain four stacking models, namely AA-S1, AA-S2, AA-O1, AA-O2, and AB-S1, AB-S2, AB-O1, AB-O2, respectively. Similarly, the AC family consists of AC-S1, AC-S2, AC-O1, and AC-O2. Here, the labels S and O indicate that the sulfur-terminated or oxygen-terminated surface of the Janus HfSO monolayer faces the GeCH layer, whereas the indices 1 and 2 distinguish different in-plane atomic registries within the same interface orientation.

**Fig. 2:**
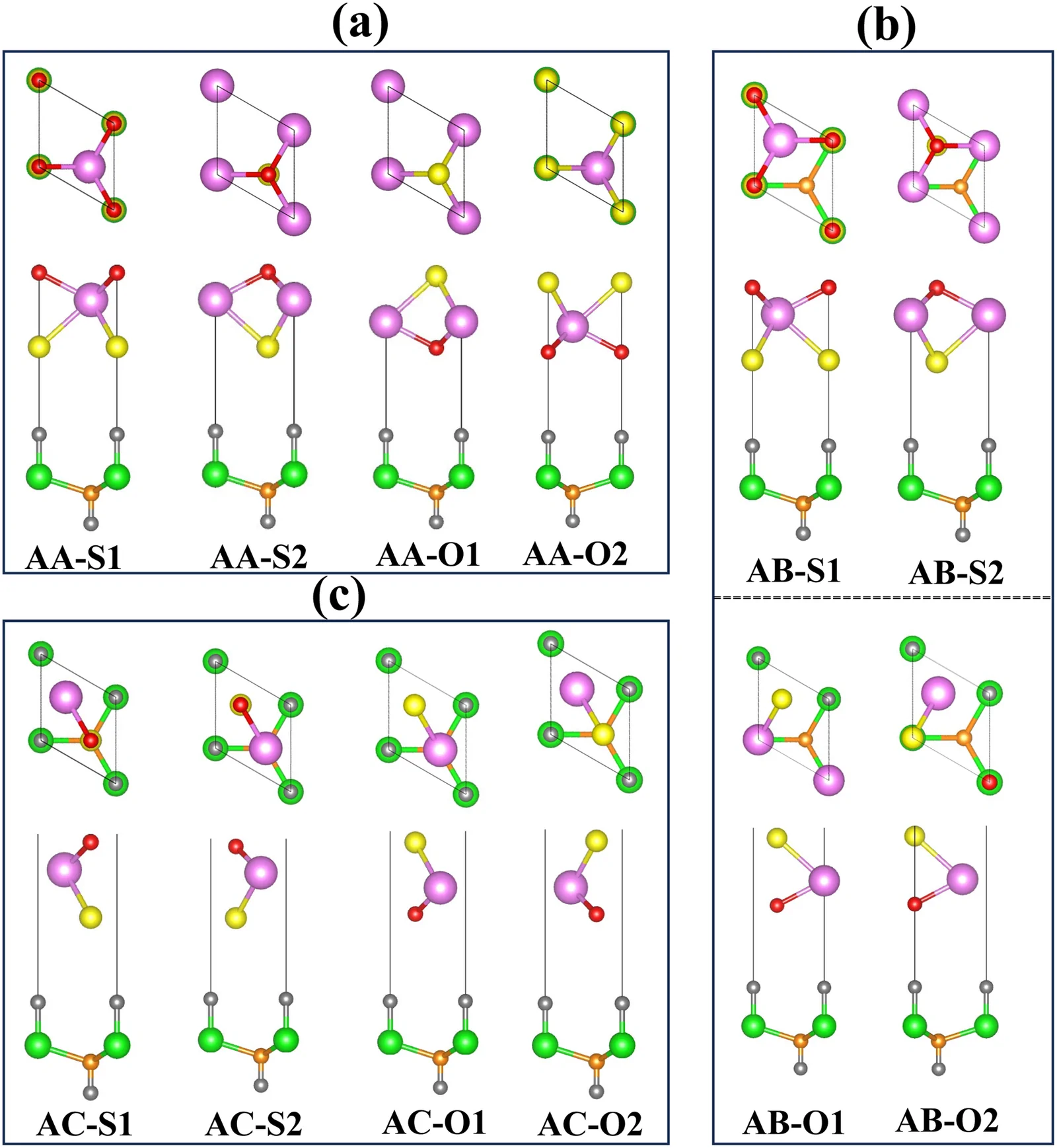
Schematic illustration of the twelve initial stacking configurations considered for the GeCH/HfSO van der Waals heterostructure. Three representative stacking families, namely (a) AA, (b) AB, and (c) AC, were constructed by combining different in-plane atomic registries with the S-terminated and O-terminated surfaces of the Janus HfSO monolayer. Here, the labels S and O indicate that the sulfur-terminated or oxygen-terminated surface of the Janus HfSO monolayer faces the GeCH layer, whereas the indices 1 and 2 distinguish different in-plane atomic registries within the same interface orientation. The purple, yellow, red, green, orange, and gray spheres denote Hf, S, O, Ge, C, and H atoms, respectively.

Furthermore, the optimized interlayer separation and binding energy provide important indicators of the nature and strength of the interaction between the GeCH and HfSO layers. As summarized in Table 1, the equilibrium interlayer distances fall within a relatively narrow range of 3.05–3.30 Å, depending on the stacking registry and the exposed surface of Janus HfSO. The S-terminated configurations generally exhibit shorter interlayer separations of 3.05–3.20 Å, whereas the O-terminated configurations show slightly larger values of 3.29–3.30 Å. Such interlayer distances are consistent with those commonly reported for 2D vdW heterostructures. The binding energy was calculated to evaluate the energetic favorability of forming the GeCH/HfSO heterostructure and to compare the relative stability of the different stacking configurations. It is defined as:

1 *E*_b_ = *E*_GeCH/HfSO_ − *E*_GeCH_ − *E*_HfSO_,

where *E*_GeCH_, *E*_HfSO_, and *E*_GeCH/HfSO_ are the total energies of the isolated GeCH monolayer, isolated HfSO monolayer, and GeCH/HfSO heterostructure, respectively. A negative *E*_b_ indicates that the formation of the heterostructure is energetically favorable. As summarized in Table 1, all considered stacking configurations exhibit negative binding energies, confirming that the formation of the GeCH/HfSO interface is energetically favorable. The calculated *E*_b_ values range from approximately −0.21 to −0.34 eV, while the corresponding interlayer distances vary from 3.05 to 3.30 Å. The O-terminated configurations generally show larger binding energies than their S-terminated counterparts, suggesting stronger interfacial attraction at the O-facing surface. Among all considered structures, the AC-O1 configuration exhibits the lowest binding energy of −0.34 eV together with an interlayer separation of 3.29 Å, identifying it as the most energetically favorable stacking configuration. Notably, the interlayer separations of approximately 3.1–3.3 Å, together with the moderate binding energies and the absence of interlayer covalent bonds, are characteristic of noncovalent interlayer interactions. These features support the description of the GeCH/HfSO interface as the vdW heterostructure rather than a chemically bonded layered system.

**Table 1:** Calculated interlayer separation (*d*, Å), binding energy (*E*_b_, eV), band gap (*E*_g_, eV) of the GeCH/HfSO heterostructure for all considered stacking configurations

GeCH/HfSO	*d*	*E*_b_	*E* ^PBE^_g_	Nature	Band alignment
AA-S1	3.20	−0.22	0.05	Indirect	Type-II
AA-S2	3.05	−0.24	0.06	Indirect	Type-II
AA-O1	3.29	−0.31	0.83	Indirect	Type-II
AA-O1	3.30	−0.29	0.78	Indirect	Type-II
AB-S1	3.20	−0.21	0.05	Indirect	Type-II
AB-S2	3.05	−0.23	0.06	Indirect	Type-II
AB-O1	3.29	−0.30	0.83	Indirect	Type-II
AB-O2	3.30	−0.28	0.77	Indirect	Type-II
AC-S1	3.05	−0.25	0.06	Indirect	Type-II
AC-S2	3.05	−0.26	0.06	Indirect	Type-II
AC-O1	3.29	−0.34	0.82	Indirect	Type-II
AC-O2	3.29	−0.32	0.83	Indirect	Type-II

Furthermore, to elucidate the influence of the interfacial atomic registry on the electronic characteristics of the GeCH/HfSO heterostructure, the projected band structures of all twelve stacking configurations were calculated within the PBE approximation, as shown in Fig. 3. A common feature of all stacking configurations is that the GeCH/HfSO heterostructures preserve their semiconducting nature after vertical assembly. No interface-induced metallic states appear around the Fermi level, indicating that the electronic characteristics of the constituent monolayers remain largely intact upon heterostructure formation. More importantly, the projected band structures clearly demonstrate that the electronic states near the band edges originate predominantly from one individual layer, while only negligible orbital hybridization is observed around the Fermi level. The absence of pronounced anti-crossing behavior between the projected bands further confirms that the interaction between GeCH and Janus HfSO is governed primarily by weak vdW forces rather than chemical bond formation across the interface. Such weak interlayer coupling is advantageous because it preserves the intrinsic electronic properties of each constituent while allowing the interfacial electronic structure to be modulated through band alignment. Furthermore, the GeCH/HfSO heterostructure exhibits the indirect semiconductors with the VBM at the *Γ* point and the CBM at the *M* point for all twelve representative stacking families. Although the overall band dispersions remain nearly unchanged among the twelve configurations, slight variations in the band-edge positions can still be identified. These variations become more noticeable when the contacting surface of the Janus HfSO monolayer changes from the S-terminated side to the O-terminated side, whereas different lateral registries within the same interface orientation produce only minor modifications. The S-terminated heterostructures exhibit ultranarrow band gaps of approximately 0.06 eV, whereas the O-terminated counterparts possess significantly larger band gaps of about 0.84 eV, as listed in Table 1. The pronounced difference between the S- and O-terminated configurations indicates that the electronic structure is governed not only by the stacking registry but also strongly by the orientation of the Janus HfSO layer. This termination dependence can be correlated with the intrinsic polarity of HfSO layer. For the isolated Janus HfSO monolayer, the calculated work functions of the S- and O-terminated surfaces are 7.03 and 5.70 eV, respectively, yielding a substantial difference of 1.33 eV. Such a work-function asymmetry reflects the inequivalent electrostatic environments of the two surfaces and results in different interfacial potential landscapes when HfSO is coupled with GeCH. Consequently, reversing the exposed termination modifies the relative positions of the GeCH- and HfSO-derived band edges and their interfacial electronic coupling, leading to markedly different band gaps of approximately 0.05–0.06 eV for the S-terminated configurations and 0.77–0.83 eV for the O-terminated configurations. These results demonstrate that the electronic properties of the GeCH/HfSO heterostructure are highly sensitive to the orientation of the Janus layer. Another noteworthy feature is that the electronic states contributing to the VBM and CBM remain spatially localized on different constituent layers for all stacking configurations. Such electronic characteristics suggest that interlayer charge transfer can occur upon photoexcitation. This spatial localization provides a favorable condition for the separation of photogenerated carriers, which is an important prerequisite for type-II photovoltaic architectures.

**Fig. 3:**
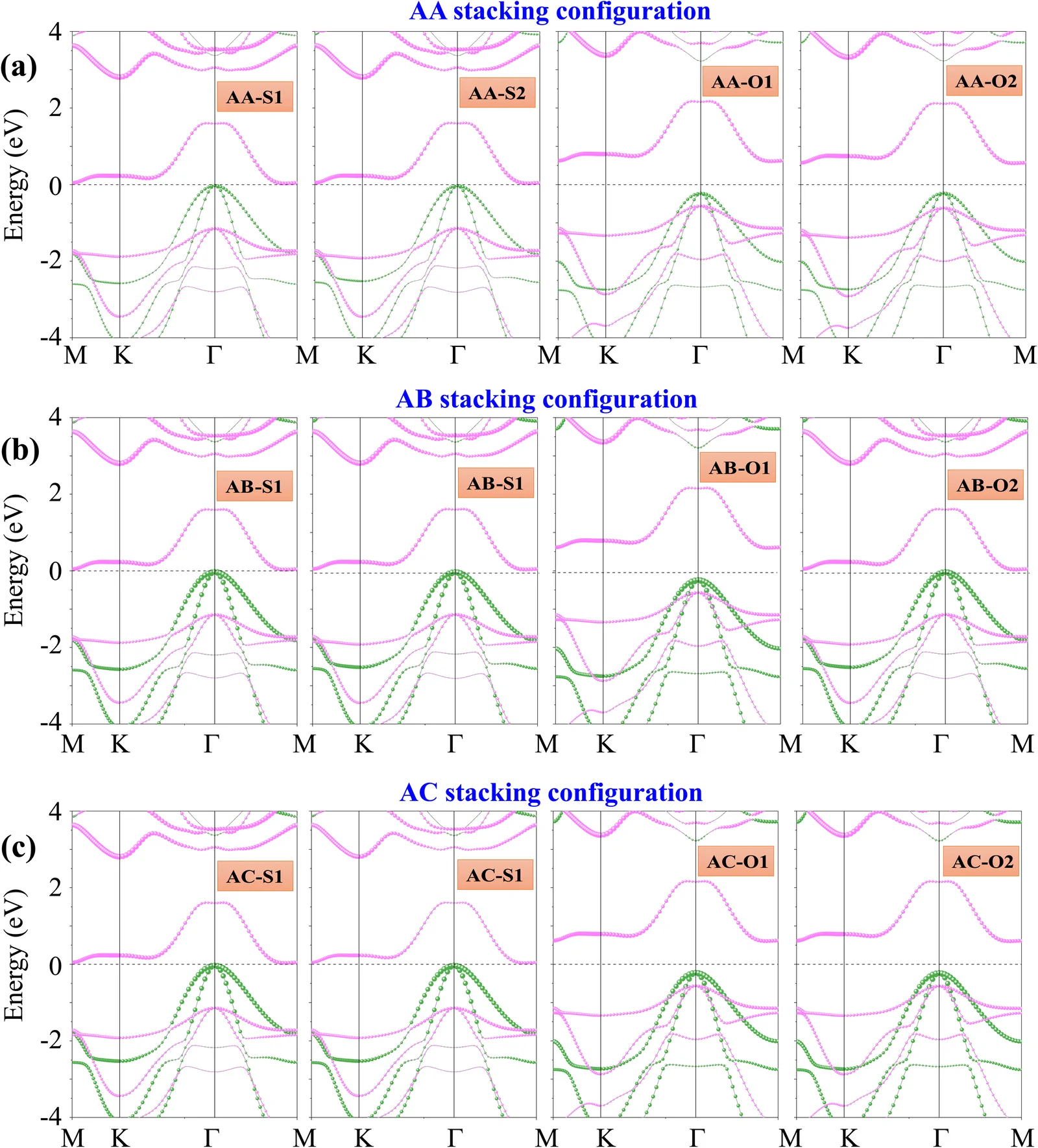
Projected band structures of three representative stacking families (a) AA, (b) AB, and (c) AC of the HfSO/GeCH heterostructure. Green and purple spheres represent the contributions of the GeCH and HfSO layers, respectively.

To further verify the electronic characteristics of the most stable AC-O1 stacking family, the projected band structure was recalculated using the HSE06 hybrid functional. As shown in Fig. 4(a), the AC-O1 heterostructure remains semiconducting after applying the hybrid functional. The calculated HSE06 band gap is 1.59 eV, which is significantly larger than the corresponding PBE value of 0.84 eV, reflecting the improved treatment of exchange interactions by the hybrid functional. Nevertheless, the overall band dispersion and the orbital contributions near the band edges remain essentially unchanged, indicating that the PBE functional reliably captures the intrinsic electronic characteristics of the GeCH/HfSO heterostructure. The projected band structure reveals that the VBM is mainly contributed by the GeCH layer, whereas the CBM originates predominantly from the Janus HfSO layer. As illustrated schematically in Fig. 4(b), this layer-resolved distribution of the band-edge states confirms the formation of a type-II band alignment, in which electrons and holes are spatially localized in different constituent layers. Upon photoexcitation, electrons preferentially transfer from the GeCH layer to the HfSO layer, while holes remain in the GeCH layer, thereby promoting efficient spatial separation of photogenerated carriers and suppressing their recombination. Such an intrinsic charge-separation mechanism is highly desirable for optoelectronic and photovoltatic applications.

**Fig. 4:**
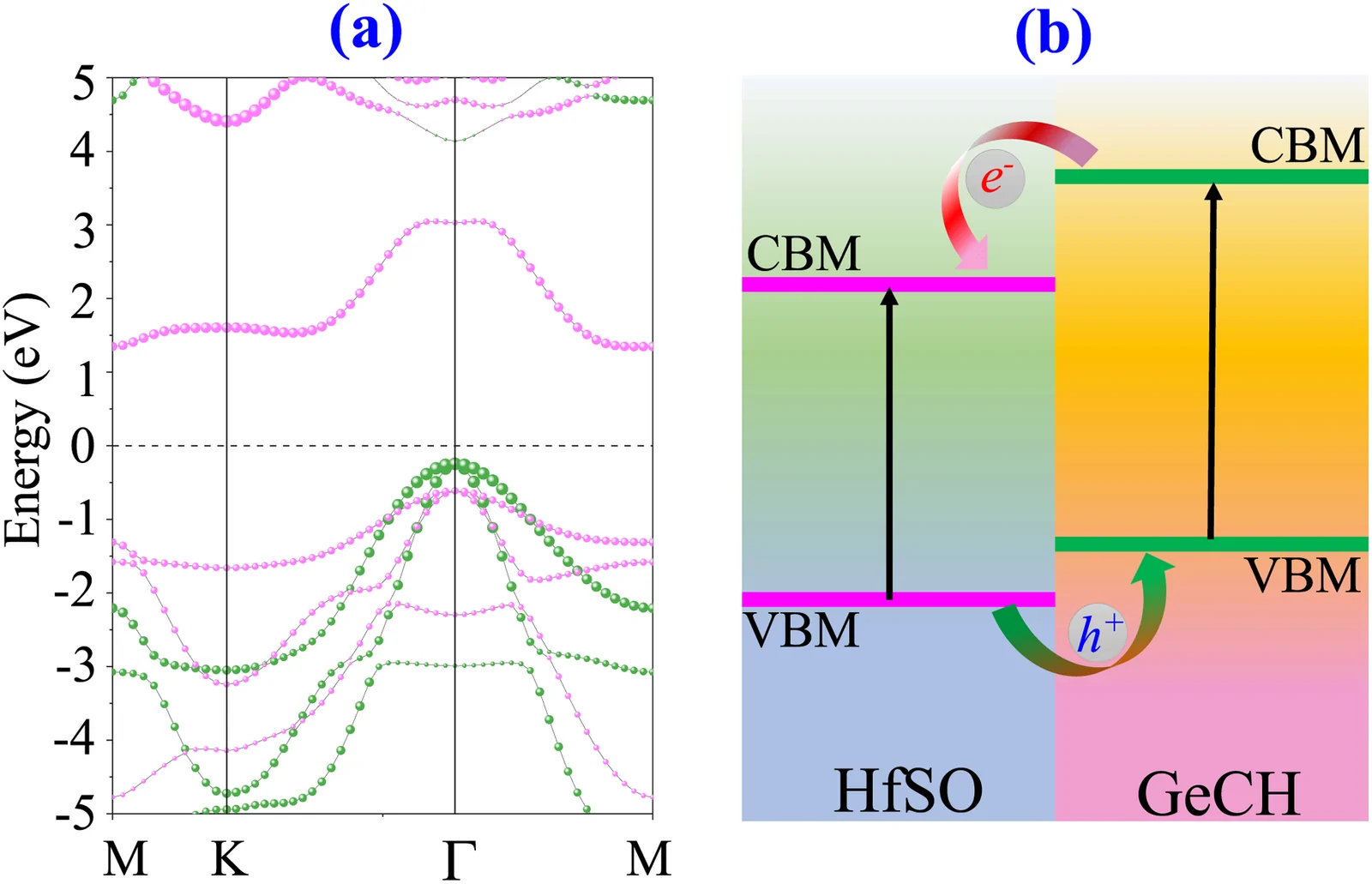
(a) Projected band structures and (b) schematic diagram of the band alignment of the most energetically stacking families AC-O1 for the HfSO/GeCH heterostructure. Green and purple spheres represent the contributions of the GeCH and HfSO layers, respectively.

The structural robustness of the optimized AC-O1 GeCH/HfSO heterostructure was comprehensively assessed by analyzing the electron localization function (ELF), phonon dispersion, and *ab initio* molecular dynamics (AIMD) simulations, as shown in Fig. 5. The ELF of the GeCH/HfSO heterostructure in Fig. 5(a) provides a quantitative description of electron localization, with values ranging from 0 to 1. An ELF value close to 1 corresponds to highly localized electrons, which are typically associated with covalent bonding or lone-pair electrons, whereas a value of 0.5 represents a nearly homogeneous electron distribution characteristic of a free-electron gas. In contrast, ELF values approaching 0 indicate electron depletion or strong electron delocalization. As illustrated in Fig. 5(a), high ELF values are mainly concentrated around the constituent atoms and along the intralayer chemical bonds, reflecting the strong covalent bonding within the GeCH and HfSO monolayers. In contrast, the interlayer region exhibits predominantly low ELF values without noticeable electron accumulation, indicating that no interfacial covalent bonds are formed after stacking. This result confirms that the two monolayers are coupled through weak vdW interactions. The dynamical stability of the optimized heterostructure was further confirmed by the phonon dispersion shown in Fig. 5(b). The absence of imaginary phonon frequencies throughout the Brillouin zone demonstrates that the AC-O1 stacking family of the GeCH/HfSO heterostructure is dynamically stable. Furthermore, the thermal stability was assessed by AIMD simulations at 300 K for 8 ps. As shown in Fig. 5(c), the total energy exhibits only small fluctuations during the simulation, and no noticeable structural reconstruction or bond breaking is observed in the final atomic structure. These results collectively confirm the excellent structural, dynamical, and thermal stability of the GeCH/HfSO heterostructure.

**Fig. 5:**
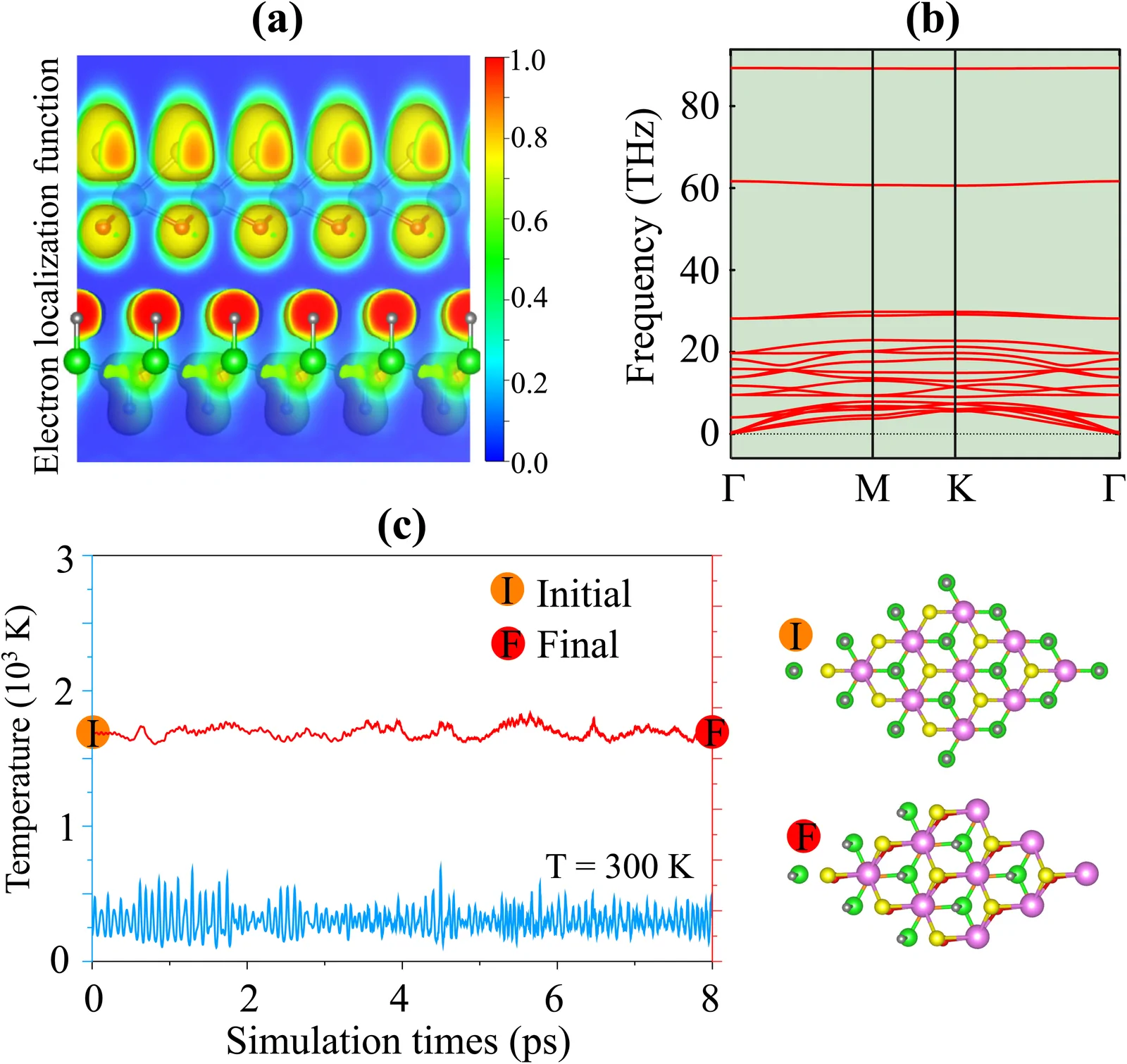
Stability analysis of the most energetically stacking family AC-O1 of the GeCH/HfSO heterostructure. (a) Electron localization function (ELF) distribution, (b) phonon dispersion, (c) evolution of the total energy during AIMD simulation at 300 K for 8 ps, together with the corresponding atomic structures at the initial (I) and final (F) simulation stages.

Furthermore, the mechanical properties of the constituent monolayers and the GeCH/HfSO heterostructure were investigated by calculating the in-plane elastic constants, as summarized in Fig. 6(a). For 2D hexagonal heterostructure, the mechanical response is fully characterized by the elastic constants *C*_11_, *C*_12_, and *C*_66_, where *C*_11_ represents the in-plane tensile stiffness, *C*_12_ describes the elastic coupling between the two in-plane directions, and *C*_66_ = (*C*_11_ − *C*_12_)/2 corresponds to the in-plane shear modulus. The calculated elastic constants of the GeCH/HfSO heterostructure are obtained to be *C*_11_ = 237.69 N m^−1^, *C*_12_ = 71.55 N m^−1^, and *C*_66_ = 83.07 N m^−1^, which satisfy the Born mechanical stability criteria, namely *C*_11_ > 0 and *C*_11_ > |*C*_12_|, confirming the mechanical stability of both the isolated monolayers and the GeCH/HfSO heterostructure. Moreover, as shown in Fig. 5(a), the GeCH/HfSO heterostructure exhibits noticeably larger elastic constants than the constituent monolayers, particularly for *C*_11_, indicating a significant enhancement in the in-plane stiffness after vertical stacking. A similar increasing trend is observed for both *C*_12_ and *C*_66_, suggesting that the formation of the vdW heterostructure improves not only the tensile resistance but also the shear rigidity. Additionally, to further evaluate the directional dependence of the mechanical response, the orientation-dependent Young's modulus was calculated according to:2
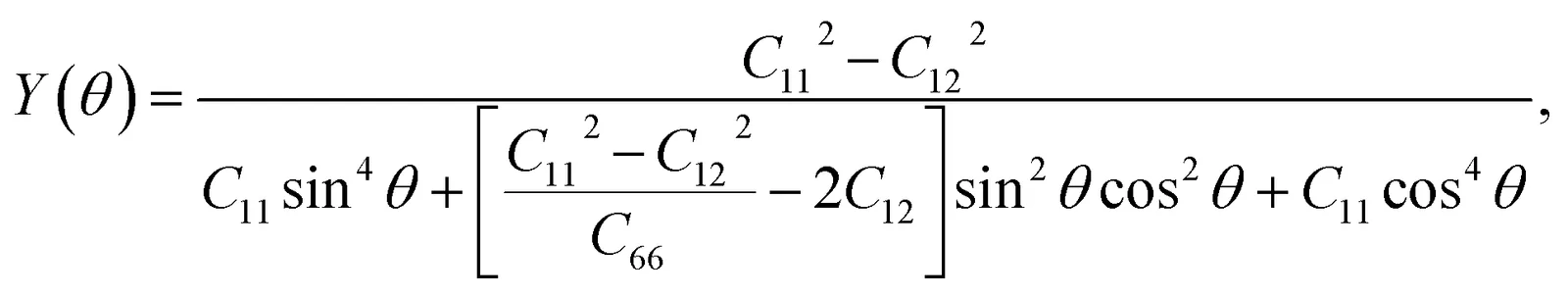
where *θ* denotes the in-plane loading direction. As illustrated in Fig. 6(b), the polar plots of Young's modulus for GeCH, HfSO, and the GeCH/HfSO heterostructure are circles, indicating isotropic in-plane mechanical behavior. Moreover, the GeCH/HfSO heterostructure possesses a substantially larger Young's modulus than the individual monolayers over the entire angular range, demonstrating that vertical stacking significantly enhances the in-plane stiffness while preserving the elastic isotropy. These results indicate that the GeCH/HfSO heterostructure combines excellent mechanical robustness with isotropic elastic properties, which are desirable for flexible electronic and optoelectronic applications.

**Fig. 6:**
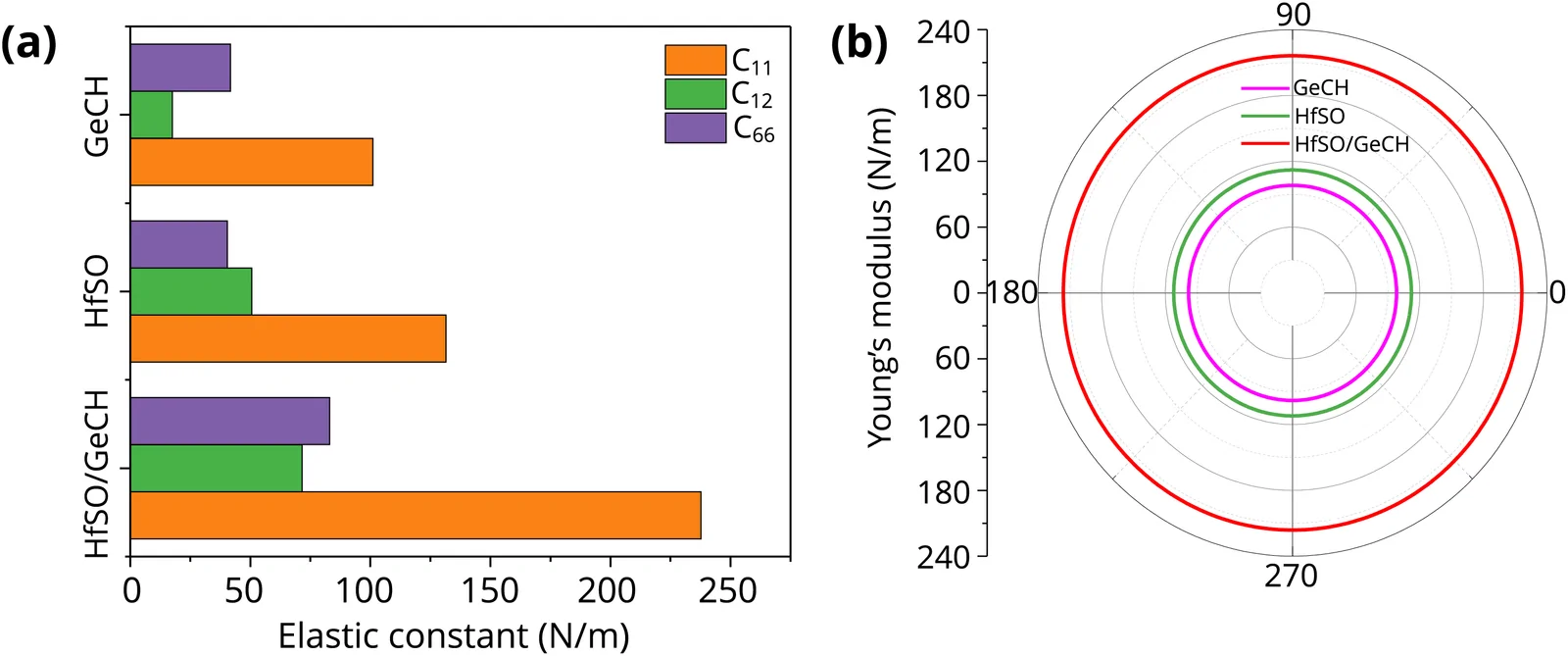
Mechanical properties of the GeCH monolayer, Janus HfSO monolayer, and the GeCH/HfSO heterostructure. (a) Calculated in-plane elastic constants (*C*_11_, *C*_12_, and *C*_66_). (b) Polar representation of the orientation-dependent Young's modulus. The radial coordinate represents the magnitude of the Young's modulus (in N m^−1^), and the angular coordinate denotes the in-plane tensile angle (*θ*).

To gain further insight into the interfacial electronic interaction, the charge density difference (CDD) and the planar-averaged electrostatic potential of the optimized AC-O1 heterostructure were calculated, as shown in Fig. 7. The CDD is defined as:

3 Δ*ρ* = *ρ*_GeCH/HfSO_ − *ρ*_HfSO_ − *ρ*_GeCH_,

where *ρ*_HfSO/GeCH_, *ρ*_HfSO_, and *ρ*_GeCH_ denote the charge densities of the heterostructure, isolated HfSO, and isolated GeCH monolayers, respectively. As illustrated in Fig. 7(a), the 3D CDD isosurface provides direct evidence of interfacial charge redistribution after the formation of the heterostructure. The cyan and pink isosurfaces correspond to electron accumulation (Δ*ρ* > 0) and electron depletion (Δ*ρ* < 0), respectively. Electron accumulation is primarily localized on the HfSO layer, whereas electron depletion is concentrated around the GeCH layer, indicating spontaneous electron transfer from GeCH to HfSO until electrostatic equilibrium is established. The planar-averaged CDD profile further confirms that the charge redistribution is mainly confined to the interface, suggesting that only a small amount of charge is transferred between the two monolayers. The planar-averaged electrostatic potential shown in Fig. 7(b) further reveals a pronounced potential gradient across the interface. The intrinsic out-of-plane asymmetry of the Janus HfSO monolayer generates a built-in electric field directed from the GeCH layer toward the HfSO layer, which drives the spontaneous electron transfer observed in the CDD analysis. As illustrated in Fig. 7(b), this internal electric field effectively promotes the spatial separation of photogenerated electrons and holes, thereby suppressing carrier recombination and reinforcing the type-II band alignment.

**Fig. 7:**
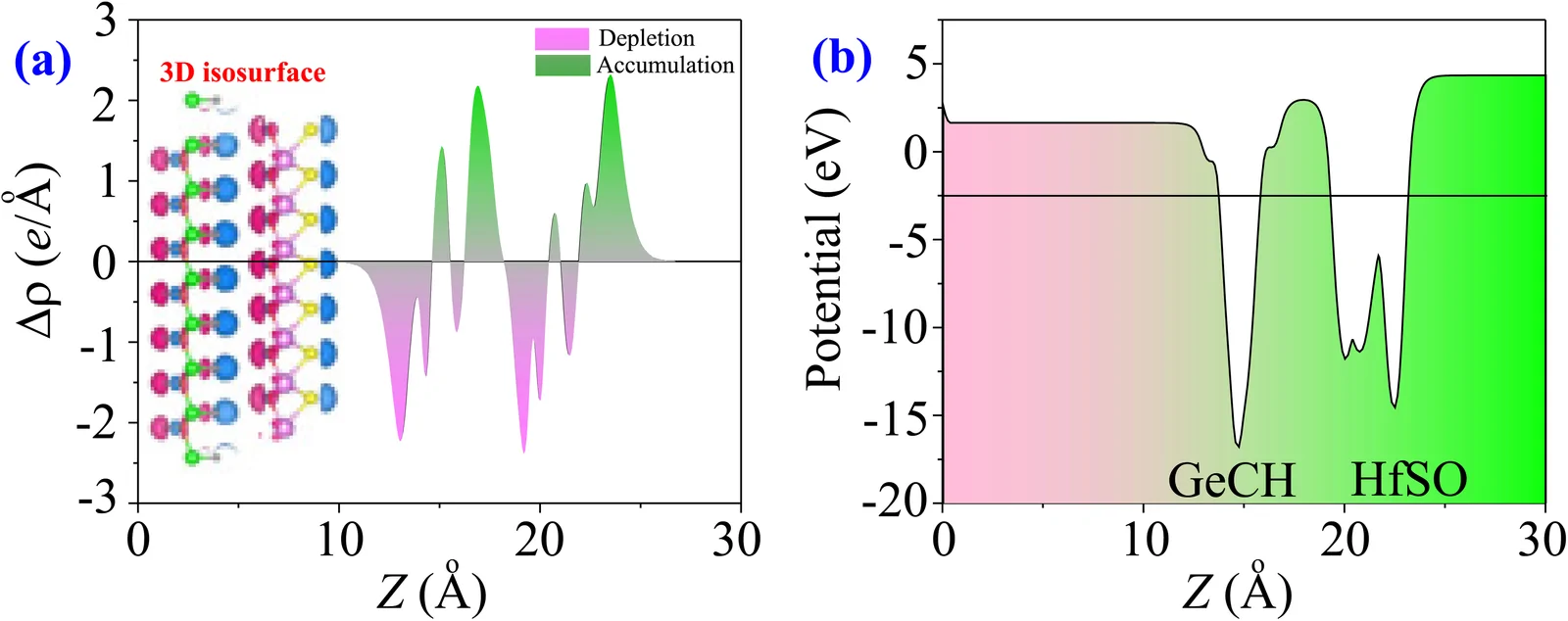
(a) 3D charge density difference (CDD) isosurface (inset) and the corresponding planar-averaged CDD (Δ*ρ*) profile along the vertical *z*-direction. For the 3D isosurface, the cyan and pink colors indicate electron accumulation and depletion, respectively. (b) Planar-averaged electrostatic potential across the heterostructure along the *z*-direction of the GeCH/HfSO heterostructure.

To assess the influence of the interfacial electronic interaction on the light-harvesting capability, the optical absorption properties of the constituent monolayers and the GeCH/HfSO heterostructure were systematically investigated. Since the device's efficiency is strongly governed by their ability to absorb incident photons, analyzing the optical response provides valuable insight into the effectiveness of heterostructure engineering for enhancing solar-energy utilization. The optical absorption coefficient was calculated from the frequency-dependent complex dielectric function,

4 *ε*(*ω*) = *ε*_1_(*ω*) + i*ε*_2_(*ω*)

where *ε*_1_(*ω*) and *ε*_2_(*ω*) denote the real and imaginary parts of the dielectric function, respectively. The absorption coefficient is given by:5
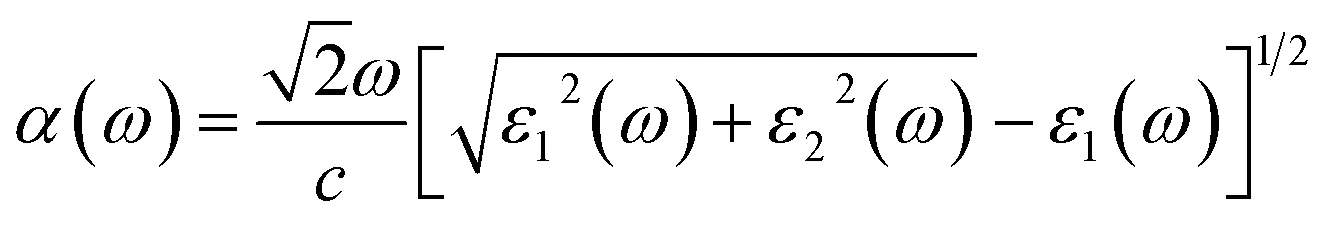
where *ω* is the photon angular frequency and *c* is the speed of light in vacuum. Fig. 8 compares the calculated optical absorption spectra of the GeCH monolayer, Janus HfSO monolayer, and the GeCH/HfSO heterostructure together with the AM1.5G solar spectrum. The GeCH monolayer exhibits negligible absorption throughout most of the visible-light region because of its relatively large band gap, whereas the Janus HfSO monolayer displays a much stronger optical response with an absorption onset extending into the visible range. After forming the GeCH/HfSO heterostructure, the absorption coefficient is markedly enhanced over a broad photon-energy range and reaches the order of 10^5^ cm^−1^. More importantly, the enhancement is most pronounced within the energy range where the AM1.5G solar spectrum exhibits its highest photon flux, indicating a more efficient utilization of solar radiation. This improvement can be attributed to the reduced band gap and the interfacial electronic coupling introduced by the type-II band alignment, which enables additional optical transitions compared with the isolated monolayers. Consequently, the GeCH/HfSO heterostructure exhibits superior light-harvesting capability, highlighting its potential for high-performance optoelectronic and solar-energy conversion applications.

**Fig. 8:**
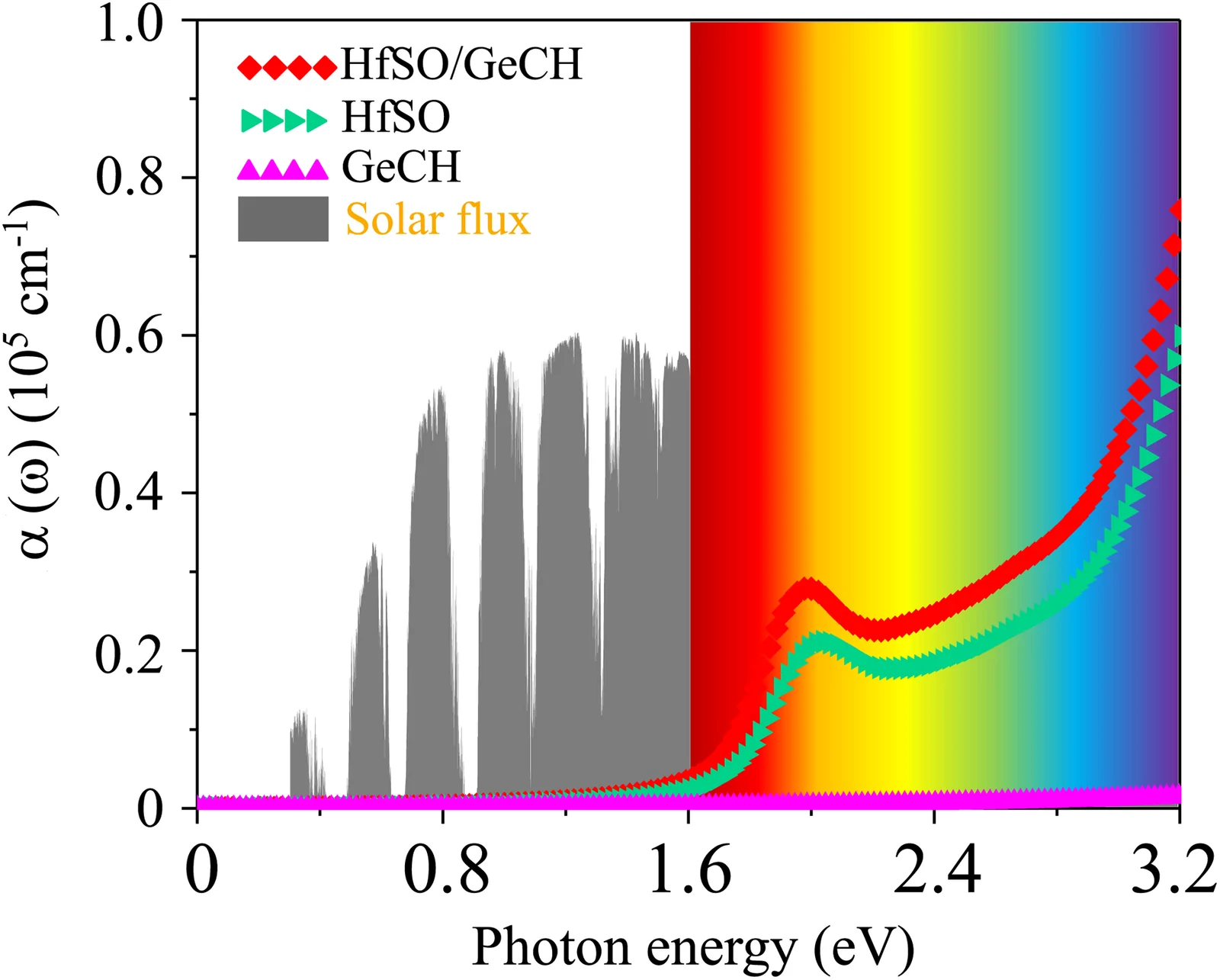
Optical absorption coefficients of the GeCH monolayer, Janus HfSO monolayer, and the GeCH/HfSO heterostructure. The visible-light region is highlighted by the colored background, and the standard AM1.5G solar photon flux (gray shaded area) is included to evaluate the solar-light harvesting capability of the GeCH/HfSO heterostructure.

Strain engineering has emerged as one of the most versatile and experimentally feasible approaches for modulating the electronic properties of 2D materials without altering their chemical composition. Such a strategy is particularly attractive for vdW heterostructures, where even moderate lattice deformation can significantly influence the interfacial electronic coupling and the relative positions of the band edges. Therefore, investigating the strain-dependent electronic properties is essential for evaluating the feasibility of mechanically tuning the performance of the GeCH/HfSO heterostructure. To explore the strain tunability of the GeCH/HfSO heterostructure, biaxial strain was applied over the range from −10% to +10%. For each imposed biaxial strain, the in-plane lattice constants were fixed at the corresponding strained values, whereas all internal atomic coordinates were fully relaxed. The out-of-plane lattice dimension was kept sufficiently large to maintain the vacuum region and avoid spurious interactions between periodic images. Before investigating the effects of strain, we first evaluated the structural stability of the GeCH/HfSO heterostructure through phonon dispersion and AIMD simulations, as shown in Fig. S1 of the SI. The absence of imaginary phonon modes confirms its dynamical stability, while the relatively small fluctuations in the total energy during the AIMD simulation indicate good thermal stability at the room temperature. The applied strain is defined as:6
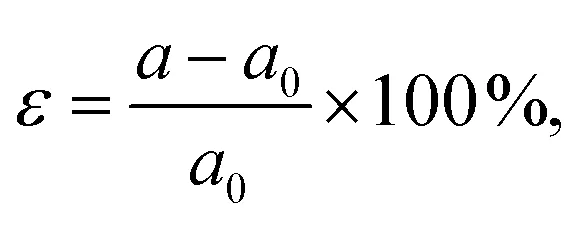
where *a*_0_ and *a* denote the equilibrium and strained lattice constants, respectively. Positive and negative values of *ε* correspond to tensile and compressive strain, respectively. Fig. 9(a) illustrates the evolution of the band gap of the GeCH/HfSO heterostructure under biaxial strain *ε*. Throughout the investigated strain range from −10% to +10%, the heterostructure preserves its semiconducting character, indicating that the electronic structure remains robust against external lattice deformation. Nevertheless, the band gap exhibits a pronounced strain dependence. Under compressive strain, the band gap decreases rapidly from approximately 0.83 eV at *ε* = 0% to about 0.19 eV at *ε* = −10%. Conversely, tensile strain gradually enlarges the band gap, reaching a maximum value of nearly 0.90 eV at around *ε* = +6%, followed by a slight reduction at larger tensile strains. To clarify the underlying mechanism, the evolution of the band-edge positions of the constituent monolayers is presented in Fig. 9(b). The VBM of the GeCH layer remains nearly insensitive to strain, whereas its CBM shifts markedly toward higher energies under compressive strain and toward lower energies under tensile strain. In contrast, both the VBM and CBM of the Janus HfSO layer exhibit relatively moderate variations under strains. Consequently, the relative positions of the band edges can be effectively manipulated by biaxial strain, leading to a tunable band alignment. More importantly, a strain-induced transition from type-II to type-I band alignment is observed. Under sufficiently large compressive strain of *ε* ≤ −8%, both the VBM and CBM are localized within the HfSO layer, corresponding to a type-I heterostructure. As the applied strain increases, the CBM of GeCH shifts upward relative to that of HfSO, while the VBM remains dominated by GeCH. This crossover of the conduction-band edges drives the transition to a type-II band alignment, which is maintained throughout the tensile strain region. Such strain-controlled band alignment demonstrates that biaxial strain provides an efficient degree of freedom for manipulating interfacial charge separation and tailoring the optoelectronic properties of the GeCH/HfSO heterostructure.

**Fig. 9:**
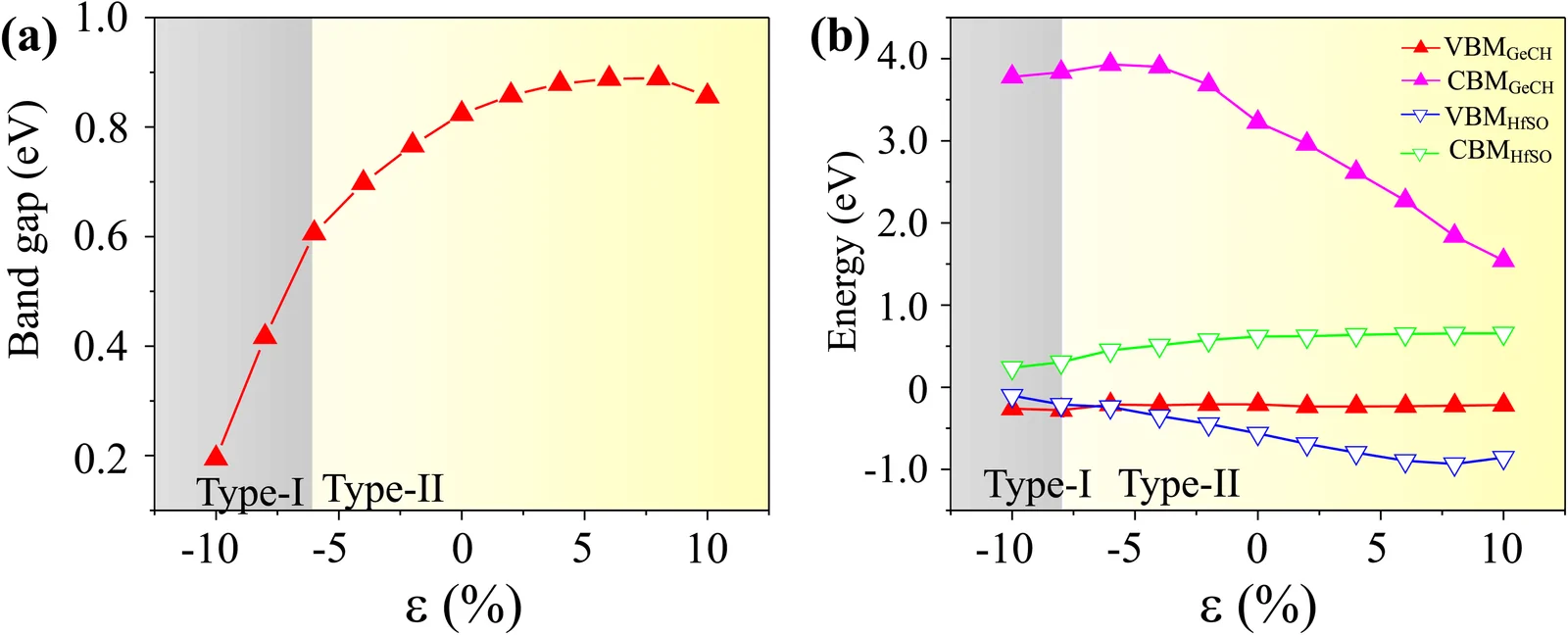
Evolution of (a) the band gap and (b) band edge positions of the GeCH/HfSO heterostructure under biaxial strain *ε*.

To gain direct insight into the strain-induced evolution of the electronic structure, the projected band structures of the GeCH/HfSO heterostructure under different biaxial strains are presented in Fig. 10. Consistent with the results shown in Fig. 9, the heterostructure retains its semiconducting character throughout the investigated strain range without undergoing a semiconductor-to-metal transition. Nevertheless, the band-edge states exhibit a pronounced dependence on the applied strain. Under compressive strain [Fig. 10(a)], the conduction-band states associated with the GeCH layer gradually shift toward higher energies as the compressive strain increases. When the strain reaches *ε* ≤ −8%, the VBM becomes localized on the HfSO layer, while the CBM remains dominated by the HfSO layer, giving rise to a type-I band alignment. It indicates that the HfSO-derived VBM shifts upward relative to the GeCH-derived VBM, leading to a crossover of the valence-band edges near *ε* ≈ −8%, where the band alignment changes from type-II to type-I. In contrast, under tensile strain [Fig. 10(b)], the CBM is consistently contributed by the HfSO layer, whereas the VBM remains localized on the GeCH layer. Although both the conduction and valence bands undergo gradual energy shifts with increasing tensile strain, the layer-resolved band-edge characteristics remain essentially unchanged, indicating that the type-II band alignment is preserved. Notably, the strain-induced evolution is governed primarily by the relative positions of the valence-band edges, particularly the crossover between the GeCH- and HfSO-derived VBMs. Consequently, tensile strain stabilizes the staggered band alignment, which is favorable for efficient interfacial charge separation. These findings demonstrate that the electronic properties of the GeCH/HfSO heterostructure can be effectively tuned through biaxial strain. In particular, the crossover between the GeCH- and HfSO-derived valence-band edges provides the physical origin of the strain-induced transition from type-II to type-I band alignment, offering an effective strategy for tailoring the electronic and optoelectronic performance of the heterostructure.

**Fig. 10:**
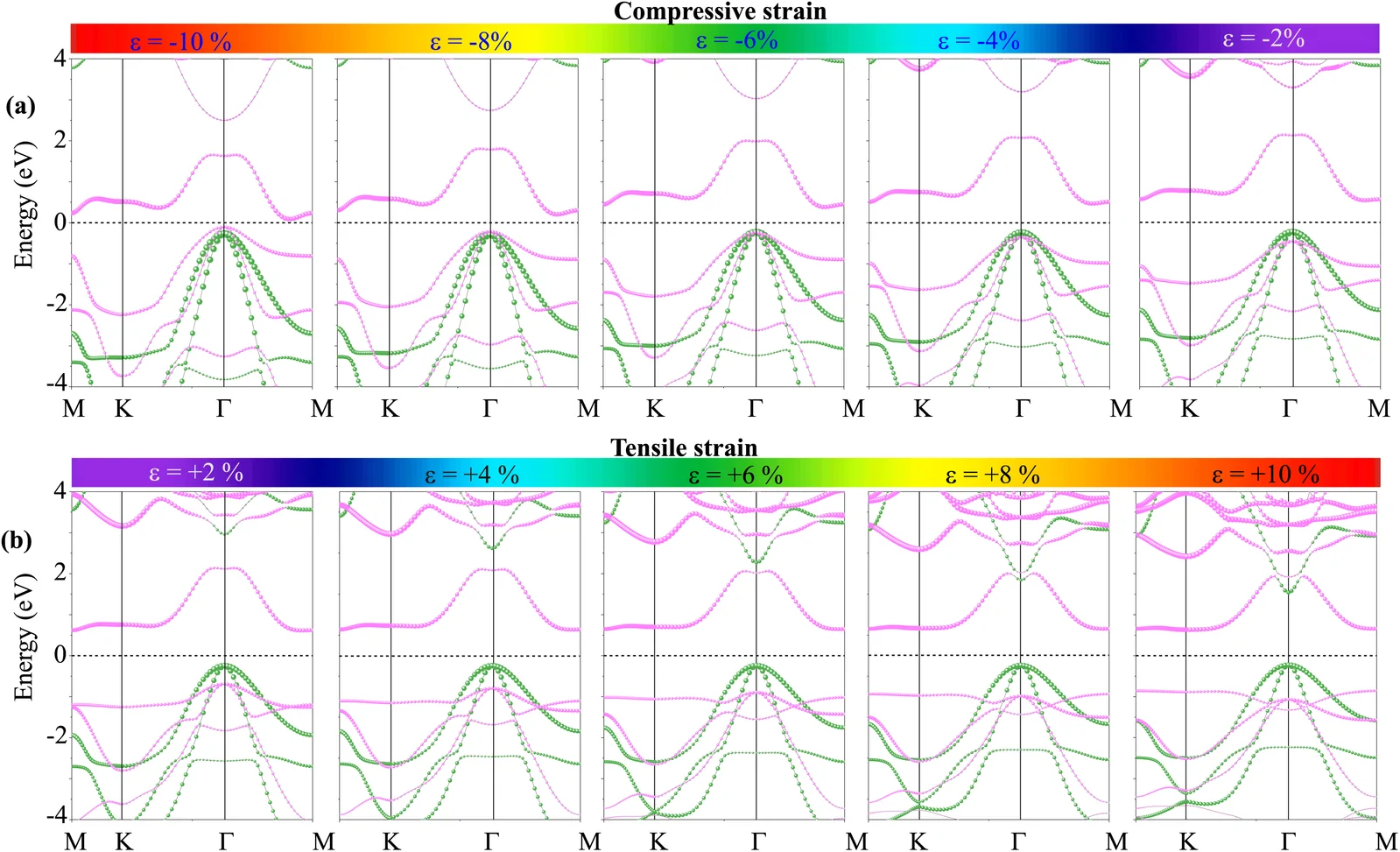
Projected electronic band structures of the GeCH/HfSO van der Waals heterostructure under different biaxial strains. (a) Compressive strain and (b) tensile strain. The Fermi level is set to zero and indicated by the horizontal dashed line. The green and magenta colors denote the orbital contributions from the GeCH and Janus HfSO monolayers, respectively.

To further assess the photovoltaic potential of the GeCH/HfSO heterostructure, the theoretical power conversion efficiency (PCE) was estimated based on the model originally proposed by Bernardi and Scharboer^47,48^ adapted for 2D vdW heterostructures. Within this framework, the maximum achievable PCE can be expressed as7
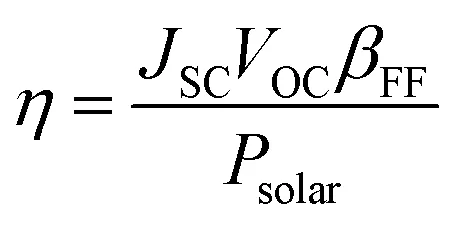
where *J*_SC_ is the short-circuit current density, *V*_OC_ is the open-circuit voltage, *β*_FF_ is the fill factor, and *P*_solar_ denotes the incident solar power under the standard AM1.5G solar spectrum. Following previous theoretical studies, the fill factor is assumed to be *β*_FF_ = 0.65. The short-circuit current density is determined from the AM1.5G solar spectrum according to:8
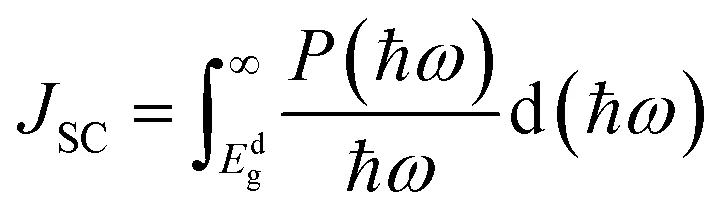
where *P*(ℏ*ω*) represents the AM1.5G solar irradiance as a function of photon energy, and *E*^d^_g_ is the band gap of the donor layer. The open-circuit voltage is approximated by:

9 *V*_OC_ = *E*^d^_g_ − Δ*E*_C_ − 0.30

where Δ*E*_C_ is the conduction-band offset between the donor and acceptor layers, while the empirical loss of 0.30 eV accounts for the energy loss during charge separation and carrier collection. Combining these quantities, the PCE can be evaluated as:10
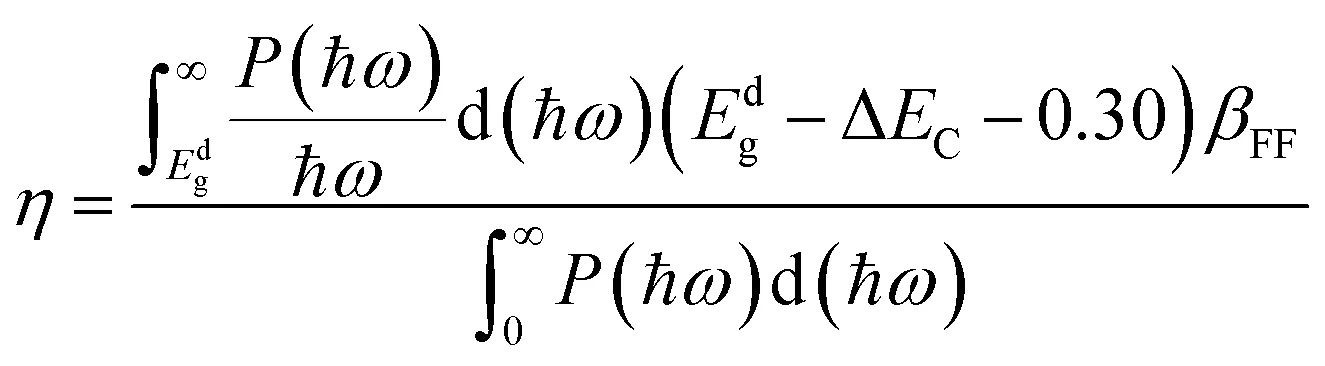


The strain-dependent photovoltaic performance of the GeCH/HfSO heterostructure was further evaluated by estimating the power conversion efficiency (PCE), as shown in Fig. 11 and Table S1 of the SI. It is evident that the pronounced enhancement in the PCE is primarily governed by the substantial increase in *J*_SC_. With increasing tensile strain from 0 to +10%, *J*_SC_ increases markedly from 5.14 to 205.06 mA cm^−2^, mainly owing to the strain-induced reduction in the donor band gap (*E*_d_) from 2.476 to 1.252 eV. At zero strain, the calculated PCE is approximately 2.23%. Upon applying tensile strain, the PCE increases progressively, reaching 4.41%, 8.24%, 13.79%, 22.60%, and 28.60% at *ε* = +2%, +4%, +6%, +8%, and +10%, respectively. Thus, a tensile strain of +10% enhances the estimated PCE by more than one order of magnitude compared with the unstrained heterostructure. It is important to emphasize that the PCE values must be explicitly defined as a theoretical estimated value based on this specific Scharber model. This theoretical framework relies on idealized assumptions, employing a fixed fill factor and an empirical energy loss parameter. It does not account for complex practical factors present in realistic operational devices, such as actual light absorption limits, exciton dissociation dynamics, non-radiative carrier recombination, non-ideal carrier transport, and electrode contact resistance. Therefore, these calculated values serve as a theoretical upper bound that highlights the intrinsic potential of the GeCH/HfSO heterostructure for solar-energy conversion.

**Fig. 11:**
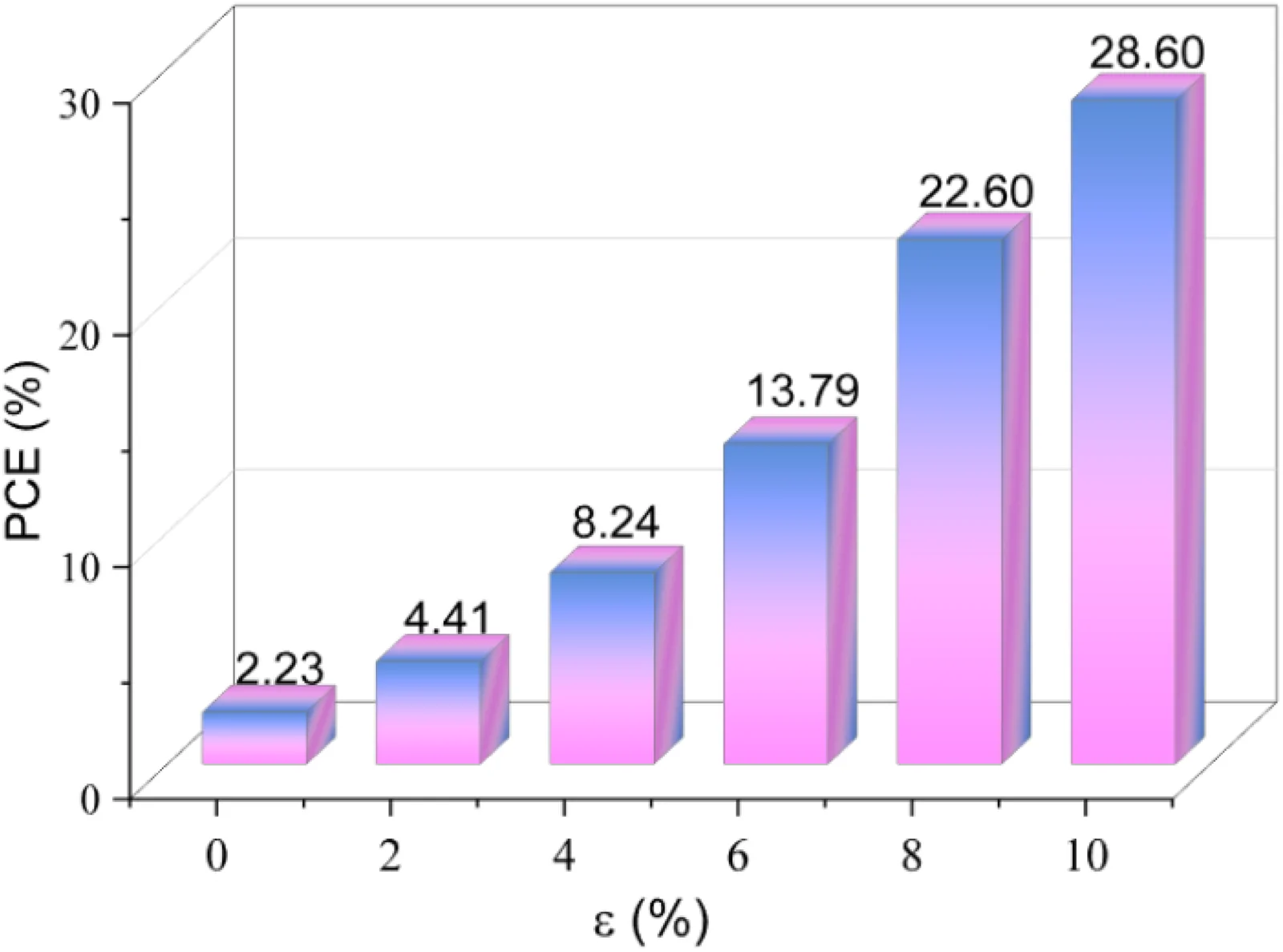
Calculated power conversion efficiency (PCE) of the GeCH/HfSO van der Waals heterostructure under tensile biaxial strain ranging from *ε* = 0% to +10%.

The pronounced enhancement in PCE can be associated primarily with the strain-dependent evolution of the band-edge alignment. As shown in Fig. 9(b), tensile strain progressively reduces the conduction-band offset between the GeCH and HfSO layers while preserving the type-II band alignment. Since the GeCH layer acts as the electron donor and the HfSO layer serves as the electron acceptor, the reduction in the conduction-band offset increases the estimated open-circuit voltage. Consequently, the more favorable band alignment under tensile strain leads to a substantial increase in the calculated PCE. This result demonstrates that mechanical strain provides an effective means of regulating the photovoltaic response of the GeCH/HfSO heterostructure, with tensile strain being particularly favorable for enhancing its solar-energy conversion performance.

## 4 Conclusions

In conclusion, we have systematically investigated the structural, electronic, interfacial, mechanical, optical, and photovoltaic properties of the GeCH/HfSO vdW heterostructure using first-principles calculations. The optimized heterostructure is characterized by weak interlayer coupling without the formation of interfacial covalent bonds, while its excellent dynamical and thermal stability is confirmed by the absence of imaginary phonon modes and the stable structural evolution during 8 ps AIMD simulations at 300 K. Moreover, the calculated elastic constants satisfy the Born mechanical stability criteria, and the enhanced Young's modulus demonstrates that the formation of the heterostructure substantially improves the in-plane mechanical stiffness while preserving isotropic mechanical behavior. More importantly, the GeCH/HfSO heterostructure exhibits a favorable type-II band alignment, in which the VBM is predominantly contributed by the GeCH layer whereas the CBM originates from the Janus HfSO layer. This staggered band alignment facilitates spatial separation of photogenerated electrons and holes, with photoexcited electrons preferentially localized on the HfSO layer and holes remaining on the GeCH layer. The heterostructure also exhibits enhanced light-harvesting capability compared with the isolated GeCH and HfSO monolayers. In particular, its optical absorption coefficient reaches the order of 10^5^ cm^−1^ over a broad photon-energy range, with pronounced absorption in the energy region corresponding to the strongest photon flux of the AM1.5G solar spectrum. These characteristics, together with the favorable type-II band alignment, demonstrate the potential of GeCH/HfSO as a promising platform for solar-energy conversion and optoelectronic applications. More importantly, the electronic and photovoltaic properties of the GeCH/HfSO heterostructure can be effectively regulated by biaxial strain. Under compressive strain of *ε* ≤ −6%, the system exhibits type-I band alignment, whereas a transition from type-I to type-II alignment occurs near *ε* ≈ −5% as the GeCH-derived CBM crosses the HfSO-derived CBM. In the tensile-strain regime, the type-II alignment is maintained, while the band gap increases. More strikingly, the theoretical estimated power conversion efficiency increases continuously from 2.23% for the unstrained heterostructure to 28.60% under 10% tensile strain. This substantial enhancement originates primarily from the strain-induced reduction of the conduction-band offset, which increases the estimated open-circuit voltage while preserving the favorable type-II band alignment. Our results establish that the GeCH/HfSO heterostructure is a promising two-dimensional platform for next-generation solar-energy conversion and optoelectronic applications.

## Conflicts of interest

The authors declare no competing financial interest.

## Supplementary Material

RA-OLF-D6RA08066A-s001

RA-OLF-D6RA08066A-s002

## Data Availability

The data that support the findings of this study are available from the corresponding author upon reasonable request. Supplementary information (SI) is available. See DOI: https://doi.org/10.1039/d6ra08066a.
