# Supplementary material for: Designing a strain-tunable hydrogenated GeCH/Janus HfSO van der Waals heterostructure for solar-energy conversion
Source: RSC Adv. 2026 Oct 23. Online ahead of print. doi: 10.1039/d6ra08066a (PMC13692005; doi:10.1039/d6ra08066a)
Supplement: Supplementary file 1 [file RA-OLF-D6RA08066A-s002.pdf]

Supplementary Information (SI) for RSC Advances.
This journal is © The Royal Society of Chemistry

Supporting Information (SI)

Designing a Strain-Tunable Hydrogenated GeCH/Janus HfSO van der Waals Heterostructure for Solar-Energy Conversion

Tran T. Nhan[1], Ngoc-Trung Nguyen[2], Trang Minh Pham[2], Tam Duy Nguyen[3], Huynh V. Phuc[4], Nguyen N. Hieu[5,6,*], Chuong V. Nguyen[7], Yoshiyuki Kawazoe[8,9], Thi Viet Bac Phung[2,10,†]

[1]Faculty of Fundamental Sciences, Hanoi University of Industry, 298 Cau Dien, Hanoi 100000, Vietnam. E-mail: tran.nhan@haui.edu.vn

[2]Center for Environmental Intelligence, VinUniversity, Hanoi 100000, Vietnam

[3]School of Chemistry, Monash University, Melbourne, Victoria 3800, Australia

[4]Division of Physics, School of Education, Dong Thap University, Cao Lanh 870000, Vietnam

[5]Institute of Research and Development, Duy Tan University, Da Nang, 550000, Vietnam. E-mail: hieunn@duytan.edu.vn

[6]Faculty of Natural Sciences, Duy Tan University, Da Nang, 550000, Vietnam

[7]Department of Materials Science and Engineering, Le Quy Don Technical University, Hanoi 100000, Vietnam

[8]New Industry Creation Hatchery Center (NICHe), Tohoku University, Sendai 980-8579, Japan

[9]Department of Centre for Inter Disciplinary Research, SRM University-AP, Amaravati 522240, Andhra Pradesh, India

[10]College of Engineering and Computer Science, VinUniversity, Hanoi 100000, Vietnam

*Corresponding authors: bac.ptv@vinuni.edu.vn; hieunn@duytan.edu

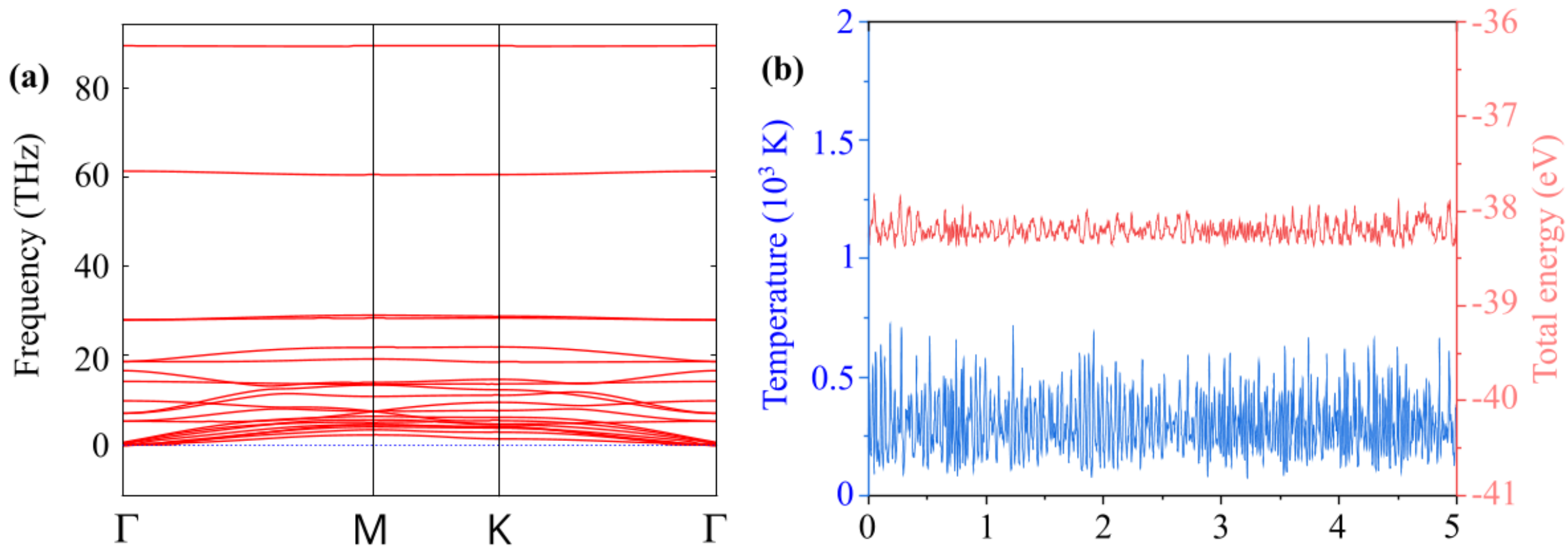

Fig. S1. (a) Phonon spectrum and (b) AIMD simulation of the GeCH/HfSO heterostructure under applied biaxially strain of +10 %.

Tab. S1. The strain-dependent parameters of the power conversion efficiency of the GeCH/HfSO heterostructure

Strain	E_d (eV)	CBO (eV)	J_{SC} (mA/cm²)	P_{solar} mA/cm²eV	PCE (%)
0%	3.445	2.476	5.14	100	2.23
+2%	3.203	2.256	10.49	100	4.41
+4%	2.86	2.081	26.49	100	8.24
+6%	2.51	1.8607	60.76	100	13.79
+8%	2.074	1.505	129.36	100	22.60
+10%	1.767	1.252	205.059	100	28.60
